# Structure-Function Relationships in *Macadamia integrifolia* Seed Coats – Fundamentals of the Hierarchical Microstructure

**DOI:** 10.1371/journal.pone.0102913

**Published:** 2014-08-07

**Authors:** Paul Schüler, Thomas Speck, Andreas Bührig-Polaczek, Claudia Fleck

**Affiliations:** 1 Materials Engineering, Technische Universität Berlin, Berlin, Germany; 2 Plant Biomechanics Group Freiburg, Botanic Garden, Faculty of Biology, University of Freiburg, Freiburg, Germany; 3 Foundry-Institute, RWTH Aachen, Aachen, Germany; Massachusetts Institute of Technology, United States of America

## Abstract

The shells/coats of nuts and seeds are often very hard to crack. This is particularly the case with *Macadamia* seed coats, known to exhibit astoundingly high strength and toughness. We performed an extensive materials science characterization of the complex hierarchical structure of these coats, using light and scanning electron microscopy in 2D as well as microCT for 3D characterization. We differentiate nine hierarchical levels that characterize the structure ranging from the whole fruit on the macroscopic scale down to the molecular scale. From a biological viewpoint, understanding the hierarchical structure may elucidate why it is advantageous for these seed coats to be so difficult to break. From an engineering viewpoint, microstructure characterization is important for identifying features that contribute to the high strength and cracking resistance of these objects. This is essential for revealing the underlying structure-function-relationships. Such information will help us develop engineering materials and lightweight-structures with improved fracture and puncture resistance.

## Introduction

Through the course of evolution, nature has developed a variety of structural and functional principles that have potential for solving problems in various fields of engineering. This concept forms the basis of biomimetic inventions and products, for example light-weight constructions with high bending resistance based on the structure of plant stems (*e.g.* [1], [2]). “Biomimetics” goes beyond “copying from nature”, by transferring functional or structural principles from biology into technology in a creative way (*e.g.* [3]).

It is well known that the shells of nuts and the coats of seeds are hard to crack despite their relatively thin walls. They have to protect the seed against deleterious environmental influences, for example UV radiation, water loss, mechanical damage when they fall or inadvertent crushing by animals seeking food [4]. The shells of *Macadamia* nuts exhibit particularly high strength values. For biologists, this poses questions about the evolutionary advantages of investing efforts in producing such high strength materials. For engineers, this makes the shell interesting as a source of inspiration for the development of impact- and puncture-resistant materials. A detailed understanding of the microstructure is the basis for identifying the structural features that are most important for bringing about the exceptional strength of these shells, and for understanding the role of their interactions during mechanical loading.

*Macadamia* nuts come from trees that have their origin in the rainforests of North-East Australia belonging to the family of Proteaceae. Of the ten known species, only *Macadamia integrifolia* and *Macadamia tetraphylla* nuts are edible and therefore economically important [5]. The individual fruits are termed follicles and they are a dehiscent type of fruit setting free the seeds which act as diaspors at ripeness. This means that no carpellate tissues are involved in the formation of the hard and tough shell. The fruits that hang in bunches of about ten when on the tree are covered by an outer leathery husk. It breaks open upon ripening such that the inner seeds – colloquially named “nuts” – become visible. Shortly afterwards, within a few days, the follicles dry out and fall down onto the ground (*e.g.* [6]).

**Figure 1:**
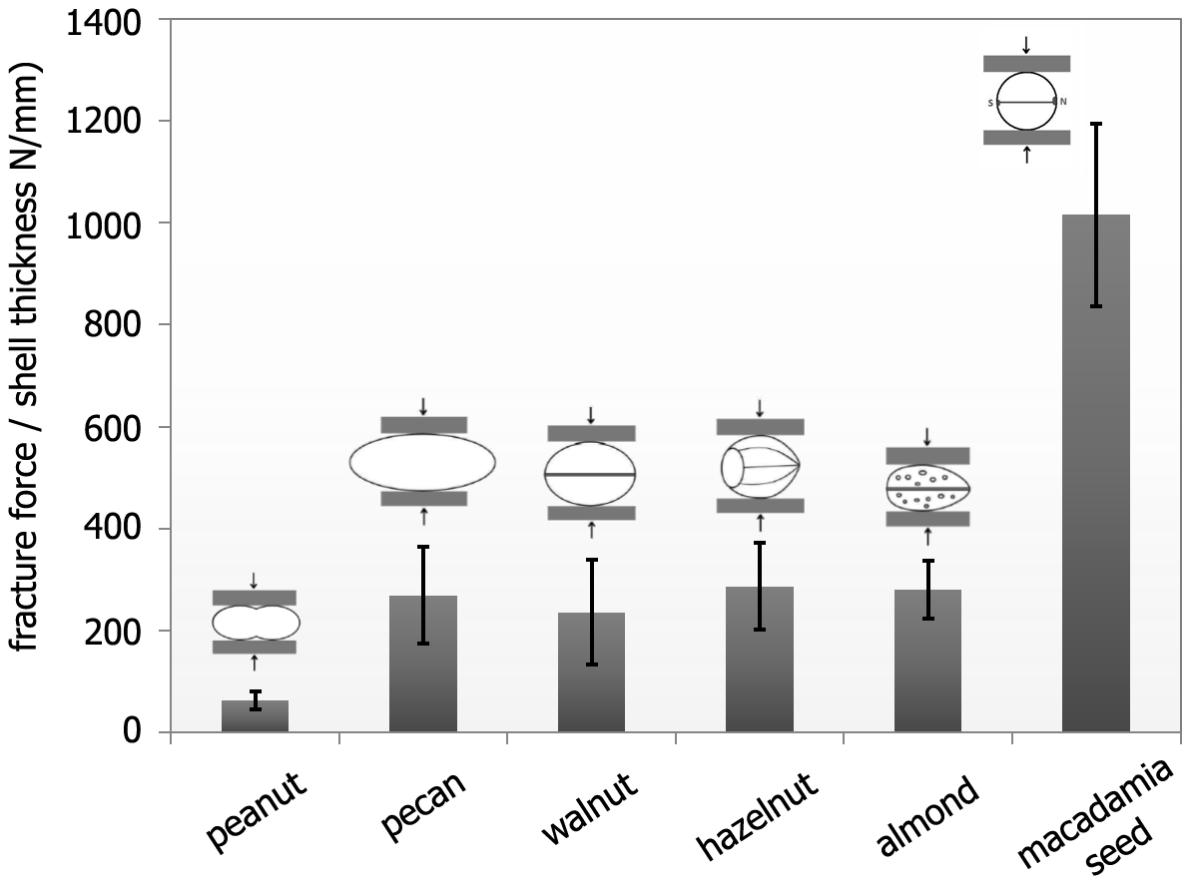
Fracture forces related to the mean shell thickness for different seed, nut and drupe shells. The sketches show the loading direction for each species. The bars are mean values, the black lines denote the standard deviations. The cross-head speed was 5 mm/min.

To break *Macadamia* seed coats, forces in the range of 1800 to 4000 N are needed: our own measurements [7]–[9] (*fig. 1* *&* *table 1*) show, in good agreement with other investigations [10]–[18] that these forces, when normalised to the shell thickness, are up to about five times higher than for other “nut” species of similar size. Our data include peanut, hazelnut, walnut and almond. The *Macadamia* seed coat material was found to exhibit the same specific tensile yield and ultimate strength (i.e. strength normalised by density) that are found in commercially pure annealed aluminium [10]. These are exceptional properties of the natural follicle and are presumably brought about by a particular structural arrangement and composition.

**Table 1:** Fracture forces of different seed coats, nut shells and hard inner drupe shells (endocarp, “fruit stone”); all specimens were tested in compression in the as-received state without additional drying with a cross-head speed of 5 mm/min.

species	shell moisturecontent (wt%)	n	Diameter(mm)	length(mm)	mean shellthickness *t* (mm)	fracture force*F*_frac_ (N)	maximum fractureforce *F*_frac, max_ (N)	*F_frac_/t* *(N/mm)*
peanut	2.0±0.5	10	15.9±0.9	47.9±1.9	1.2	76±20	117	63±17
pecan	5.5±0.9	10	23.3±1.1	44.7±3.5	1.2	323±114	433	269±95
walnut	5.1±0.3	10	28.2±0.5	35.9±1.8	1.6	378±165	72	236±103
hazelnut	5.5±0.1	10	21.7±0.3	23.4±1.3	1.5	431±127	729	288±85
almond	4.9±0.1	10	21.0±1.3	35.2±1.7	3.2	895±181	1221	280±57
*Macadamia* seed	10.1±0.7	34	23.7±1.3	23.8±1.3	2.3	2364±645	3926	1016±179

They were oriented with their sutures vertical to the loading direction and their shorter dimension parallel to the loading direction (*fig. 1*). “n” denotes the number of specimens tested. All values are mean values plus/minus standard deviations, if not denoted otherwise.

The macroscopic features of *Macadamia* and other “nuts” have been described by several authors (*e.g.* [6], [19]). Studies on the (micro−) structure and the mechanical behaviour of *Macadamia* seed coats have also been published although some are partially inconsistent or lacking in detail. For example, the shell has been described as being an “isotropic wood” by some authors [10], [12] while others stated that the shell consists of two [11], [20], [21] or three [19], [22], [23] different layers. The latter authors differentiated a 160 µm thick, hard outer zone (“outer hardshell”) and a 1500 to 5000 µm thick soft, chewable inner one (“inner hardshell”) [19].

With the exception of the innermost cell-layers, the bulk *Macadamia* seed coat has been reported to consist of sclerenchymatous cells. In contrast to most other seed coats or nut shells that are known to be basically composed of compact arrangements of sclereid cells [24], the cells in *Macadamia* seed coats have different shapes [7], [11], [20], [21]. While some authors only found fibrous cells in the shell (so-called sclerenchymatous fibres) [10], [12], others reported the presence of isodiametric cells [11], [19]–[21]. The fibrous cells have been described by some authors as being arranged in dense bundles with a random orientation [10], [12] while others report a preferred orientation [19]. Some reports noted the existence of a compact and sinuous arrangement of the single fibres without observations of bundles [11], [21]. Both the sclereids and the sclerenchymatous fibres vary in size and shape [10], [11], [21]. For the outer diameter of the fibrous cells, values in the range of 15 to 40 µm have been reported [10], [12]. The length values are less consistent: some authors report values between 100 to 150 µm [12] while others observed the fibres to have lengths up to a few millimetres [23]. The latter authors further described the existence of fine fibre bundles, oriented normal to the shell surface which they proposed act as “elastic stiffeners”. In addition to the “fullerene-like” textured surface observed, these “stiffeners” have been assumed to improve the shell resistance against external cracking [19]. Some larger voids, with diameters in the range of 100 and 400 µm have been observed [12], [21].

The cells exhibit thickened cell walls with a concentric layering [10], [12], [21]. The walls are lignified [20], [21], which increases their hardness, stiffness and strength [25]. The centre of the cells contains a rather loose material, which is assumed to be calcium oxalate [20] or lignin [11], [12].

**Figure 2:**
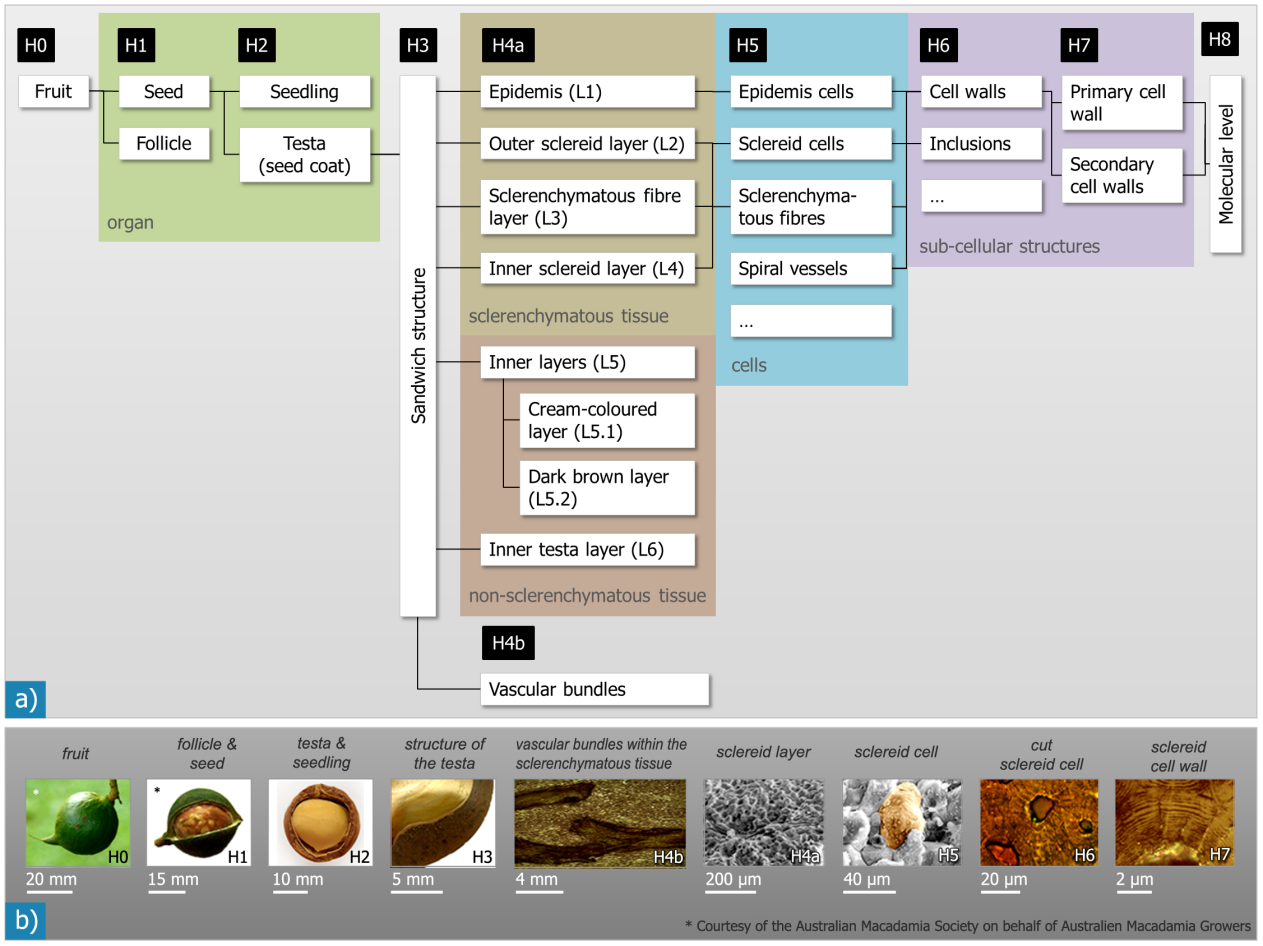
Hierarchical microstructure as observed for *Macadamia integrifolia* follicles and seeds: The overview in a) shows the different hierarchical levels (H0–H8) and the structural elements they contain. The coloured boxes link our structural levels with the corresponding classification scheme generally used in biology (organ, tissue, cells, sub-cellular structures). The images in b) give examples for structural units/features of each hierarchical level.

The present study aims at a comprehensive characterization of the hierarchical (micro−) structure of *Macadamia* seed coats. We have chosen a materials scientific classification of the hierarchical levels that is more detailed than the classification generally used in biology (*fig. 2*). As the milli- and micrometer scale of the structure are in our special focus regarding transfer to engineering materials, sub-micrometer aspects such as the cell wall organization and molecular composition have not been considered. Mechanical tests of whole seeds and specimens taken from the shells and comprising different hierarchical levels have been combined with different imaging methods [8], [9] and are currently expanded in order to evaluate the mechanical behaviour and importance of the different hierarchical levels. A detailed description of the mechanical properties at the different hierarchical levels and their contribution to the overall mechanical behaviour is beyond the scope of this paper.

## Materials and Methods

### Material

Dry *Macadamia integrifolia* seeds were supplied by Mac Nuts WA, Australia. The average moisture content was 10.1 wt% (standard deviation: 0.7 wt%), determined gravimetrically based on 10 fracture pieces of 5 seeds by weighing them before and after treatment at 105°C for at least 24 h, according to the method described by Braga and co-workers [14].

### Microscopic and tomographic investigation

**Figure 3:**
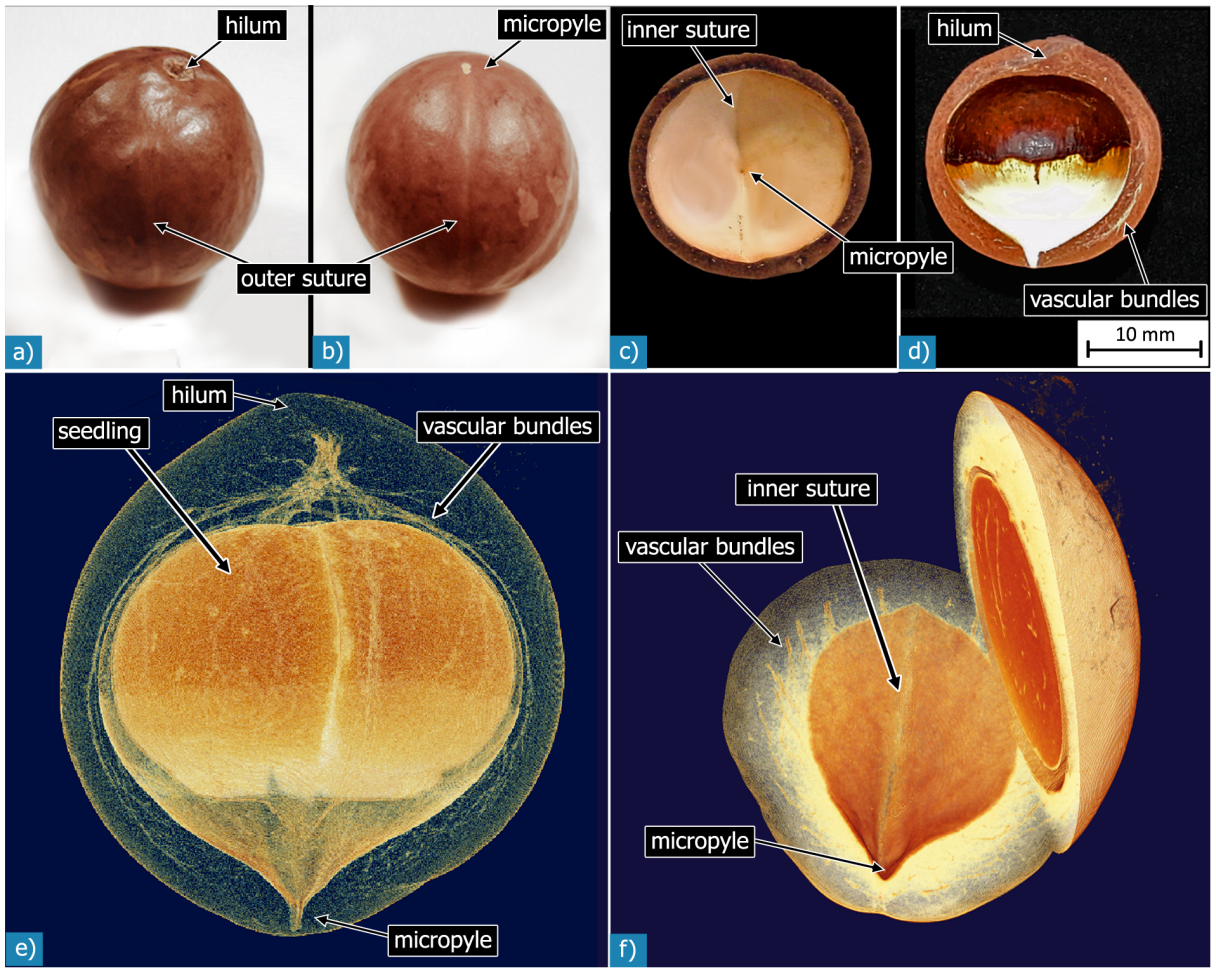
Photographs of an entire *Macadamia integrifolia* seed, showing the hilum, the micropyle and the outer suture (a, b). Light-brown speckles form an individual pattern on the surface of each shell (b). Photographs of seed coats cut normal (c) and parallel (d) to the outer suture show a locally varying thickness of the shell. The “normal” section (c) exhibits a nearly constant thickness, while the shell thickness of the “parallel” section (d) varies. The white structures within the coat material are the vascular bundles. Reconstructed 3D images from CT scans show the density, orientation and branching of the vascular bundles running from the hilum to the micropyle within the seed coat (e, f).

For microstructural investigations, coats of several seeds were cut normal and parallel to the suture, a line clearly visible on the outer surface (see results section, hierarchical level H1 and *fig. 3*). The cut shell pieces were embedded in epoxy resin and ground on SiC-paper under constant water irrigation down to a grain size of 2500 (that is 10 µm). Grinding was performed in several steps with small gradations of the abrasive particle size in order to achieve surfaces sufficiently smooth to view the microstructure of the seed coat material. In a final step, the sections were polished with a diamond suspension of 3 µm grain size on a soft cloth. The polished sections were air-dried in a desiccator for up to 7 days, avoiding the formation of drying-cracks. The sections were then observed with a Keyence VHX 100 light microscope (Keyence Deutschland GmbH, Neu-Isenburg, Germany) and, for higher magnifications, with a Leica DMRM light microscope with a MicroCam 1.3 camera (Leica Microsysteme Vertrieb GmbH, Wetzlar, Germany).

In addition to light microscopy, microstructural investigations were performed with a scanning electron microscope (SEM) CamScan CS24 (Obducat CamScan Ltd, Waterbeach, United Kingdom) on several polished sections as well as on fracture surfaces and outer surfaces of the seed coat. For these investigations, the specimens were coated with a thin gold-layer of approximately 10 to 20 nm thickness. The outer surfaces were investigated in the natural state and after dewaxing the samples by submerging whole seeds for 12 hours in a dishwashing detergent solution, followed by drying in ambient air for 2 hours as described by Kaupp & Kaupp [22].

**Figure 4:**
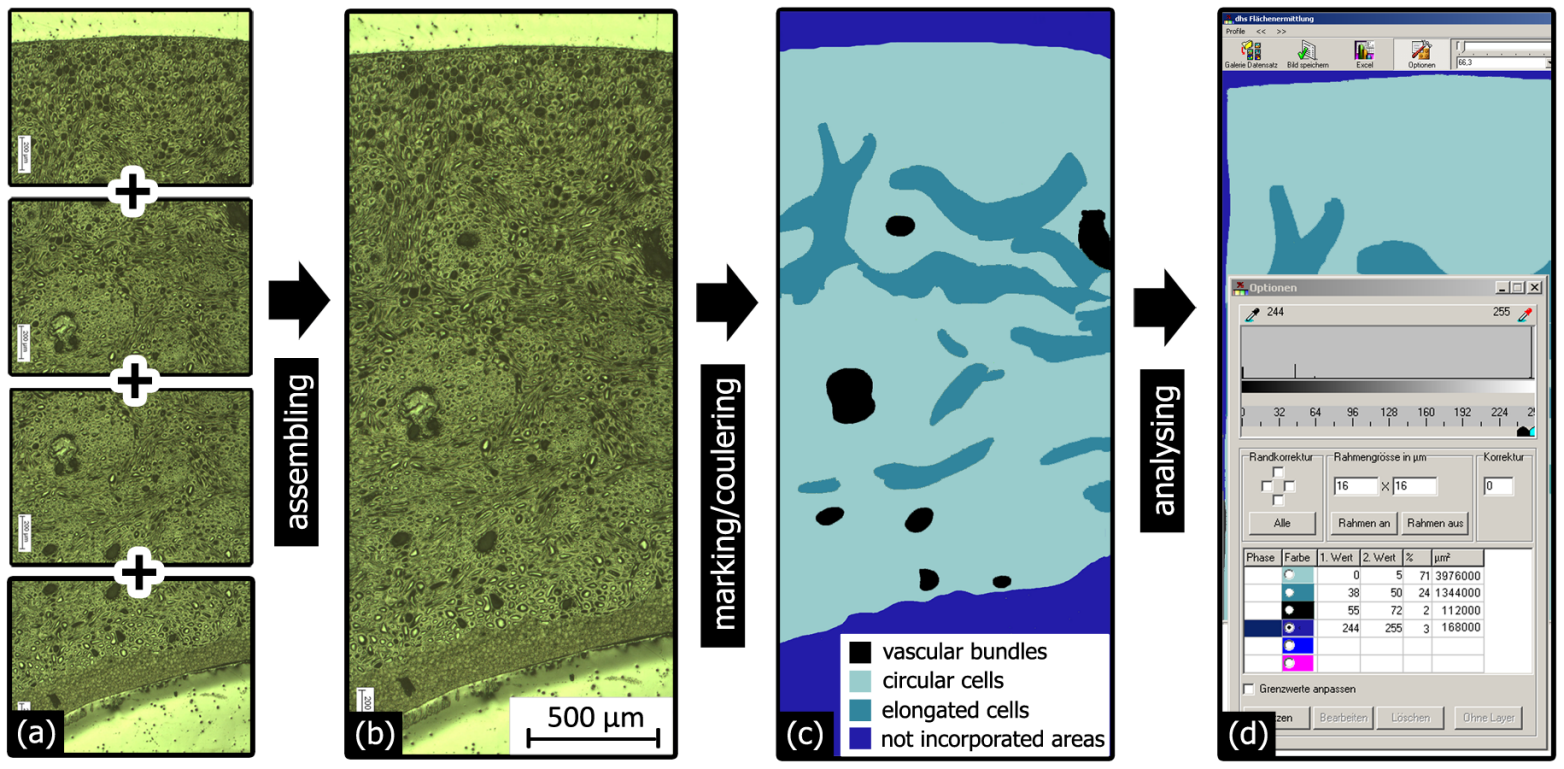
Schematic representation of how the relative area fractions of certain cell types and of the vascular bundles were analysed. Several high resolution micrographs (a) were combined (b). The different cell types and the vascular bundles were marked and colour-coded (c). The area fractions were then determined by means of a quantitative image analysis software (d).

The thickness of the different layers of the *Macadamia* seed coat was quantitatively analysed on a total of 35 micrographs of full-thickness fracture surfaces of eight different nuts. To determine whether the sclerenchymatous fibre bundles have a preferred orientation, two sections cut normal and four sections cut parallel to the suture were investigated in greater detail. The procedure of this analysis is schematically shown in *fig. 4*. In the micrographs circular and evidently elongated cells as well as vascular channels were manually marked. Features were defined as “circular” if their aspect ratio was lower than 1∶3, and as “elongated” if their aspect ratio was greater than this. The different types of marked cells/vascular bundles were each assigned to a defined colour to allow quantitative image analysis (dhs Bilddatenbank software, Mikroskop Technik Rathenow GmbH, Rathenow, Germany). Area fractions were calculated for the circular and elongated cells and the vascular bundles. The difference in area fraction of elongated cells or vascular bundles between the two cutting directions was then used as an estimate for the degree of anisotropy.

To assess the three-dimensional organisation of internal structural elements, whole nuts were scanned by microCT (Micro NDE, BAM Federal Institute for Materials Research and Testing, Berlin, Germany) with a spatial resolution of 16 µm. Following reconstruction, the 3D volume data was observed using ImageJ (Rasband, 1997–2011) and Amira 5.1 (Visage Imaging GmbH, Berlin, Germany).

## Results

The *Macadamia* seed coat exhibits a hierarchically organised microstructure. Nine main levels exist, ranging from the level of the entire fruit down to the molecular scale. An overview of the hierarchical levels that we differentiated and the correlation to the scheme usually applied in biology is shown in *fig. 2*. The structural organisation and the quantitative data determined on the different length scales is summarised in *table 2*. In biology the definition of the hierarchical levels usually follows the scheme ‘organism/organs/tissues/cells/sub-cellular structures’. Here however, we follow a materials science approach and we use other criteria for the differentiation, as often mechanical properties are linked to the microstructure. A hierarchical level was defined as a level of scale with a specific structure that is clearly discriminated from higher and lower structural levels. Entities comprising certain structural levels also have specific (mechanical) properties. The following, different levels are observed:

**Table 2:** Quantitative data (min–max value; mean ± standard deviation) describing the structural features of the *Macadamia* seed coat at the different hierarchical levels (HL) (*fig. 2*).

HL	structure	shape/structuralcomposition	diameter	length		thickness	
			(µm) unless otherwise stated				
H0	entire fruit	spherical	25–40 mm		–		–
H1	seed	spherical	22–27 mm		–		–
H2	shell (testa)	hollow sphere with varying wall thickness	–		–		1.0–4.0 mm
	seedling	almost spherical	12–24 mm		–		–
H3	shell structure	sandwich (5–6 layers) plus network ofvascular bundles	–		–		–
H4	outer epidermis L1	one layer of flat cells	–		–		5–10
	outer sclereid layer L2	compact arrangement of sclereids	–		–		800±370, 280–1530
	sclerenchymatous fibre layer L3	compact arrangement of tens tohundreds of fibrous cells	fibre bundle:100–400		fibre bundle: a few mm		1270±530, 460–2130
	inner sclereid layer L4	compact arrangement of sclereids	–		–		35±25, 0–90
	cream-coloured layer L5.1	arrangement of polyhe-dralnon-sclerenchyma-tous cells	–		–		170±75, 85–280
	dark brown layer L5.2	arrangement of slab-shaped cells	–		–		55±20, 15–85
	inner testa layer L6	flat, homogeneous	–		–		1–2
	vascular bundles	arrangement of spiral vessels	up to 500		a few mm		–
H5	cells (in L1)	pancake-like	20–50		20–40		5–10
	sclereids (in L2 & L4)	spherical, ellipsoidic, dumbbell- orkidney-shaped	20–50		1–3 times the diameter		–
	sclerenchymatous fibres (in L3)	fibrous	10–30		hundreds of µm		–
	cells (in L5.1)	polyhedral	20–30		30–40		–
	cells (in L5.2)	slab-shaped	10–20		20–35		–
	spiral vessels (in vascular bundles)	tubes	8–12		hundreds of µm		–
H6	cell wall (sclereids)	thickened, sandwich	–		–		7–15
	cell wall (scleren-chymatous fibres)	thickened, sandwich	–		–		7–10
	inclusion (scleren-chymatous cells)	polyhedral	5–30		–		–
	cell walls (in L5.1)	thin	–		–		1
	cell walls (in L5.2)	thickened	–		–		2–5
H7	cell wall structure(sclerenchymatous cells)	different composition or orientation ofthe mole-cular entities [26], [27]	–		–		ca. 0.1–2

### Hierarchical level H0

A whole *Macadamia* fruit in the mature state has a round shape with outer diameters of 25 to 40 mm [6].

### Hierarchical level H1

The fruit consists of an outer leathery shell, the pericarp of the fruit (follicle), and the inner seed. *Macadamia integrifolia* seeds have a spherical shape with outer diameters between 22 and 27 mm in the case of the seeds we investigated. The diffusely arranged light-brown, round or elongated speckles with diameters between 1 and 10 mm form an individual pattern for each seed coat (*fig. 3a & b*). A natural wax layer gives the surface a shiny appearance. Further macroscopic features of the seed on this level are the hilum and the micropyle. The hilum is the site of the seed where it was connected to the placenta with the follicle [*6*]. The micropyle – the entrance area of the pollen tube into the ovule wall – is located approximately opposite to the hilum. A barely visible depression, the “suture”, joins the hilum and the micropyle on the outside of the seed shell as a semi-circular line.

### Hierarchical level H2

The seed is a hollow shell-structure enclosing the seedling (*fig. 3e*). The protective hard seed coat is called the testa and surrounds the seedling or kernel. On the inner surface of the testa two regions can be distinguished: the half of the testa facing the hilum is of a dark brown colour, while the opposite hemisphere – adjacent to the micropyle – is of a light cream-colour (*fig. 3c & d*). The interface between these two differently coloured hemispheres is orientated nearly perpendicular to the outer suture line found along the greatest diameter of the seed coat.

Sutures appear also on the inside of the testa, as one or two notch-like depressions, joining the hilum and the micropyle (*fig. 3c & f*). These “inner sutures” are deepest at the micropyle, where they form a V-notch, and become shallower towards the hilum. In the dark brown hemisphere they are often only visible as thin brown-grey lines. One of the inner sutures is always situated on the inside directly opposite the outer suture, or enclosing a small angle, while the other one, which was only found in a few seeds, establishes an angle of 90° with the first one.

The thickness of the shell wall varies considerably between 1 and 4 mm depending on the specific region of measurement. The thickest values are generally found below the hilum and in the vicinity of the micropyle while the seed shell is at its thinnest at an intermediate position between the hilum and the micropyle and at the micropyle. If a seed is cut normal to the outer suture, a cross-section with an almost constant thickness is obtained (*fig. 3c*) while in sections cut parallel to the suture that contain the hilum, great variations in wall thickness are observed (*fig. 3d*).

### Hierarchical level H3

**Figure 5:**
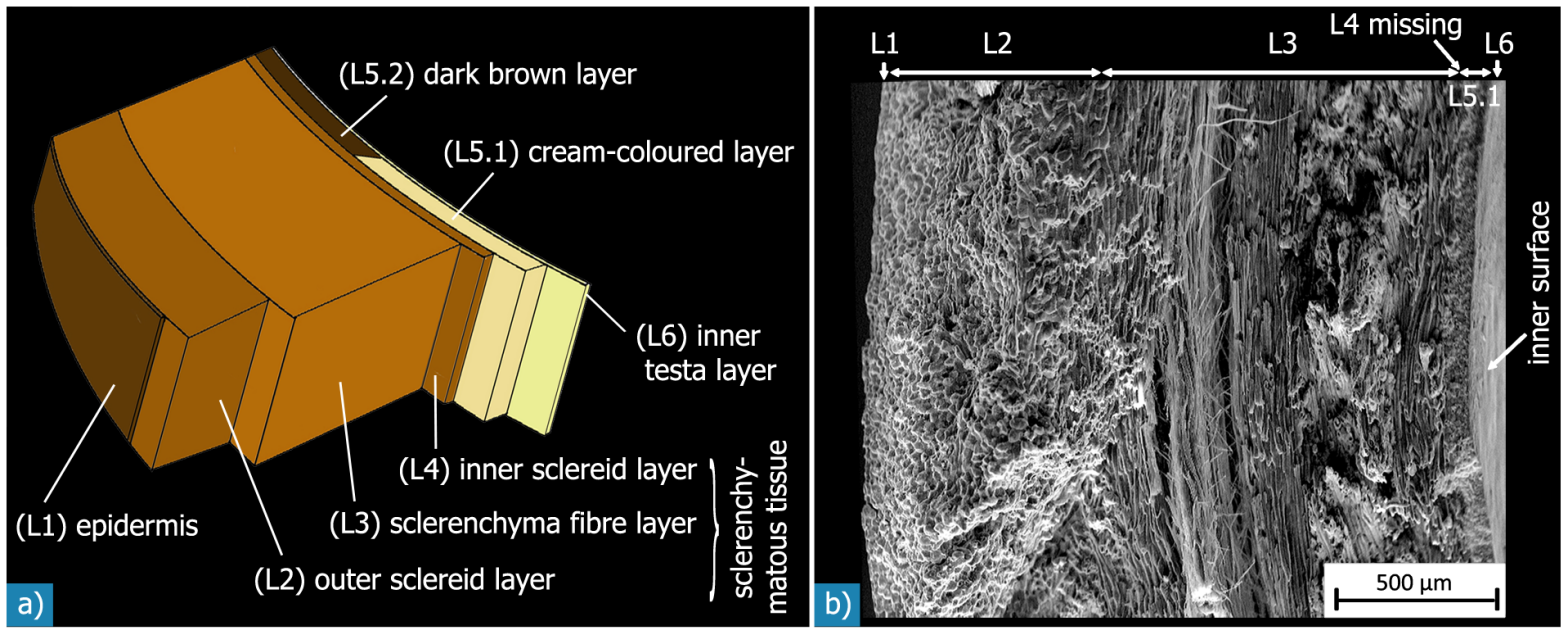
Sandwich structure of the *Macadamia* seed coat: a) schematic illustration While layers L2 to L5.1 are visible at the resolution of the SEM micrograph of a fracture surface shown in (b), layers L1 and L6 may only be discerned at higher magnifications (*fig. 6*).

The testa is a sandwich structure of concentric layers (*fig. 5*), except for the direct area of the micropyle, where it consists only of the cream-coloured tissue. The multi-layered structure of the testa was best seen by scanning electron micrographs of fracture surfaces. The number of layers varies, depending on whether we classify according to the anatomic origin or the shape of the cells: regarding the anatomic origin, four layers - the epidermis, the sclerenchymatous tissue, the cream-coloured or dark brown layer, and the inner testa layer - may be distinguished from the outside to the inside of the testa. Regarding the different prominent cell types, two to locally three (sub−) layers can further be distinguished in the sclerenchymatous tissue layer (see H4).

### Hierarchical level H4

**Figure 6:**
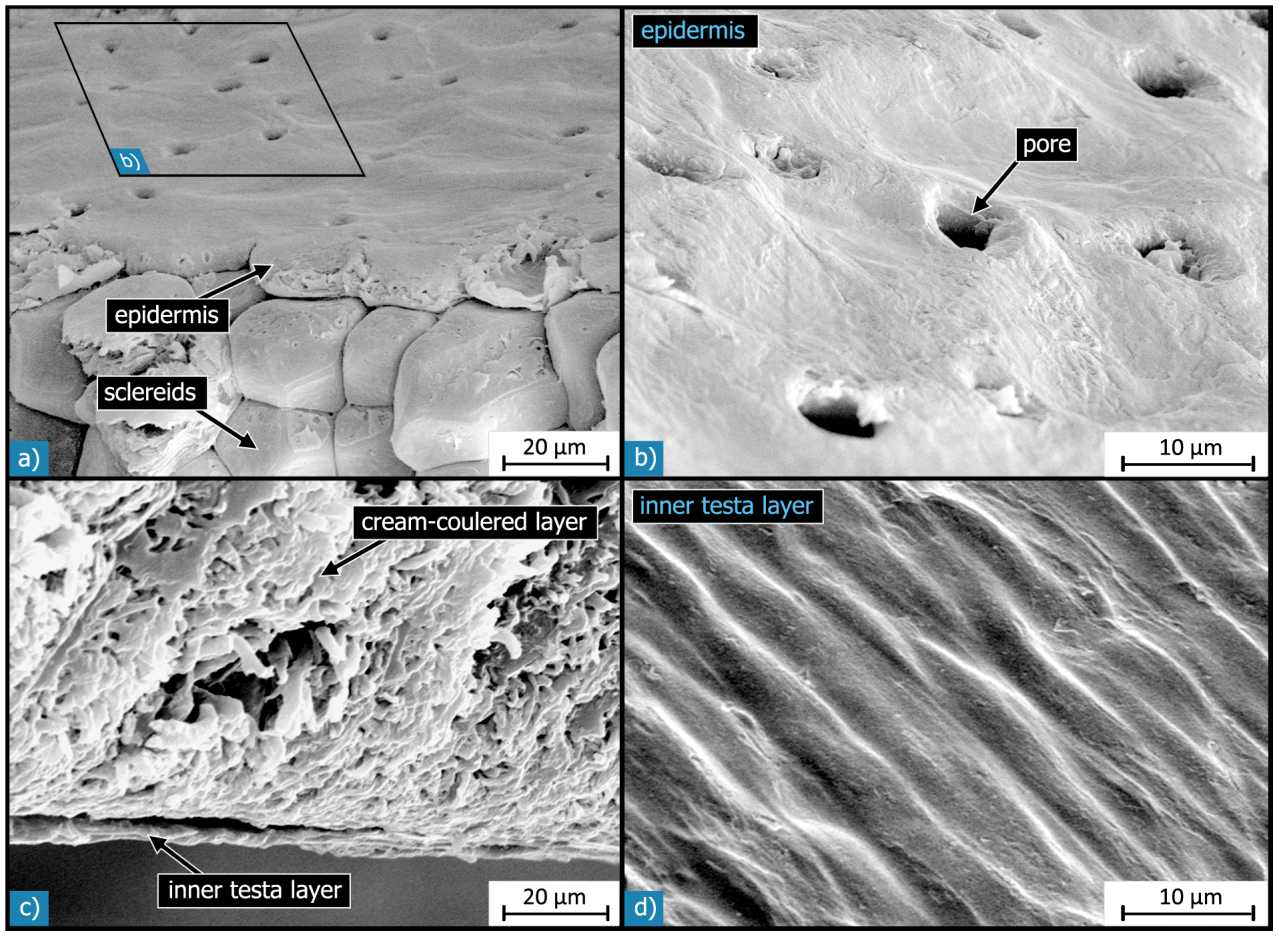
SEM micrographs of the outer- and innermost layers of the *Macadamia integrifolia* seed coat: a) & b): epidermis, c) & d) inner testa layer. The one cell-layer thick epidermis is composed of pancake-like cells, which form a smooth surface with many pores on the outside. The inner contour of the epidermis cells follows the shape of the sclereid cells. The inner testa layer is a thin homogeneous layer, which is connected to the cream-coloured or dark brown inner layers. The surface of the inner testa layer shows no pores (d).

**Figure 7:**
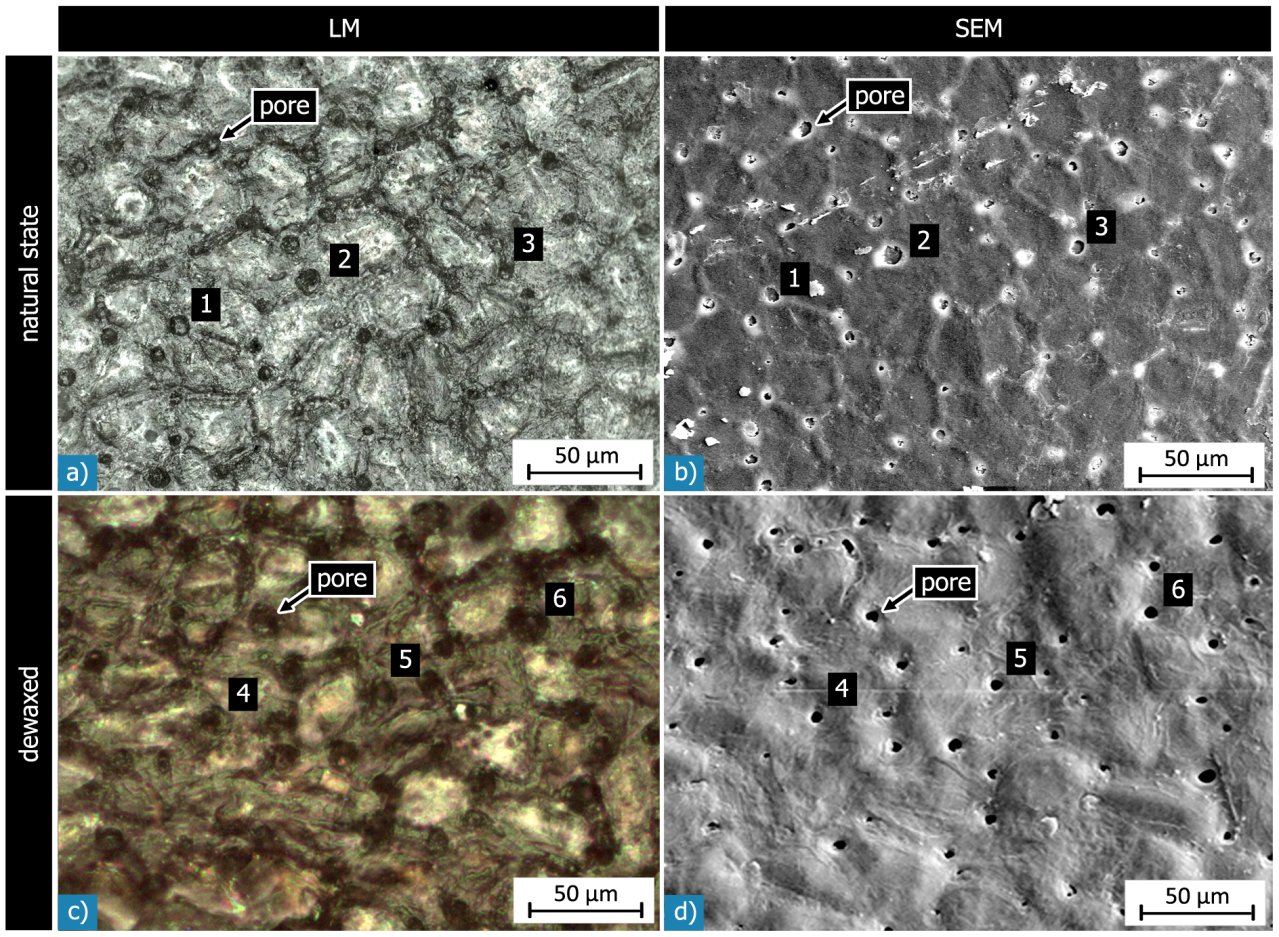
Light (LM) and scanning electron (SEM) micrographs of corresponding sections of the outer surface of the same *Macadamia* seed coat: a) & b) with natural wax layer, c) & d) after dewaxing. In both states many pores are seen on the shell’s surface. They appear as dark circular objects in the light micrographs, and are even better visible in the SEM micrographs. The arrows and the numbers denote corresponding pores in the LM and SEM micrographs. The polyhedral surface structure visible in the natural state is less well visible after dewaxing.

This level corresponds to the structural composition of the different layers and the vascular bundles. The outermost layer (epidermis, L1) consists of one cell-layer of flat pancake-like cells (*fig. 6a*). This outermost layer further features many pores that seem to be located between the individual cells and have diameters in the range of 2 to 5 µm (*fig. 6b*). By dewaxing of the surface of otherwise untreated seed coats the pores are better visible because some pores are covered by the thin wax layer (*fig. 7*). In contrast, the polygonal shape of the cells visible on the outside in the natural state becomes less well visible following dewaxing.

**Figure 8:**
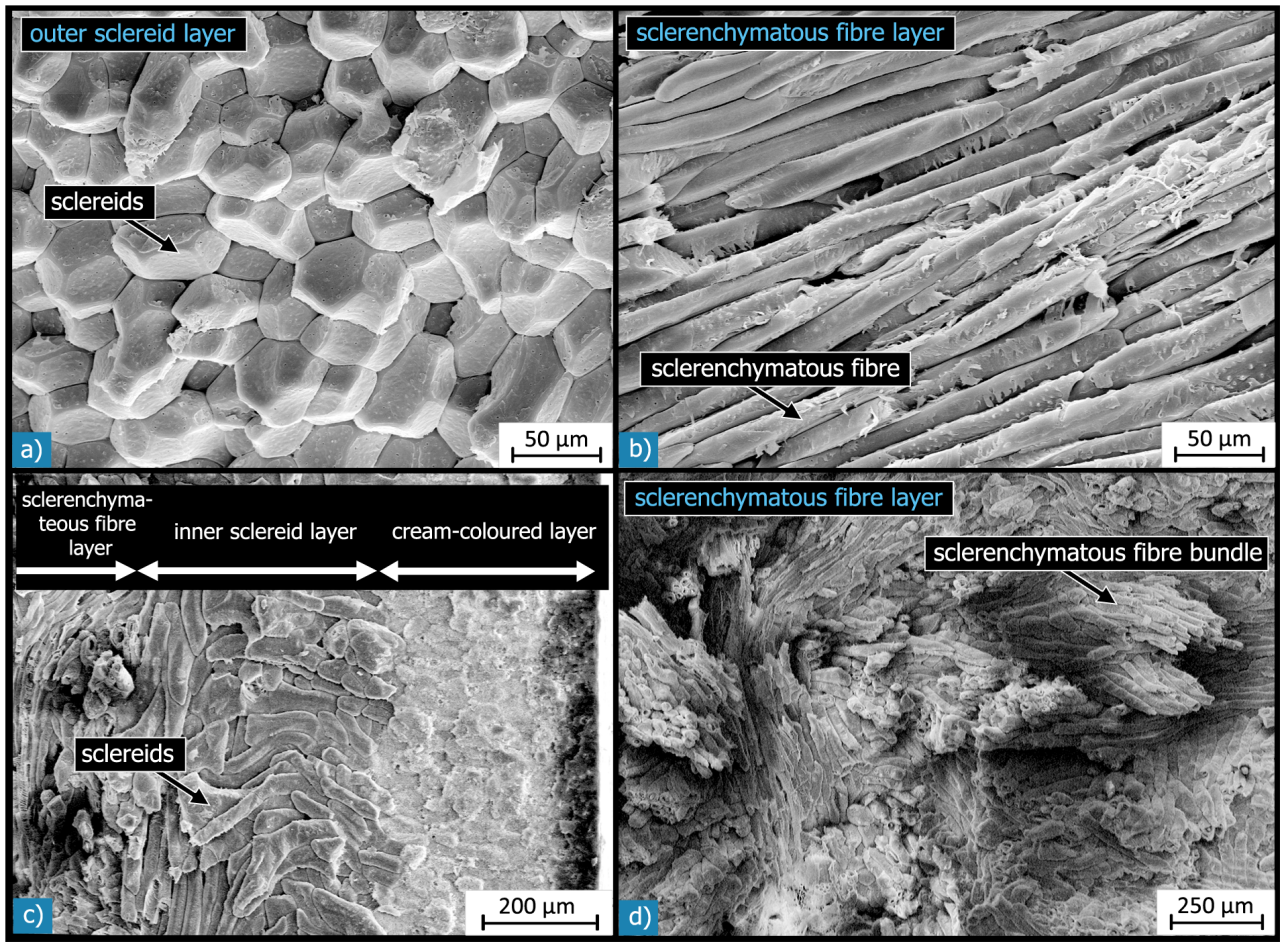
SEM micrographs of the different sclerenchymatous layers. a) Outer sclereid layer (L2), which is composed of a dense arrangement of polyhedral sclereids; b) the sclerenchymatous fibre layer (L3), which consists of fibrous cells, so-called sclerenchymatous fibres; c) in some regions of the shell, another relatively thin “inner” sclereid layer (L4) was observed, which contains ellipsoidic, kidney- or dumbbell-shaped sclereids; d) the sclerenchymatous fibres are arranged in compact bundles, which are entwined with each other.

The outer sclereid layer (L2) is about 800 µm thick, and therefore makes up about one third of the testa (*table 2*). It consists of a densely packed arrangement of polyhedral sclereids (*fig. 8a*).

The sclerenchymatous fibre layer (L3) has an average thickness of about 1300 µm, which corresponds to about 50% of the seed coat thickness (*table 2*). The sclerenchymatous fibres are arranged in compact bundles of tens or hundreds of elongated, fibre-like cells (*fig. 8b & d*). These bundles have lengths of up to a few millimetres and diameters of 100 to 400 µm.

**Figure 9:**
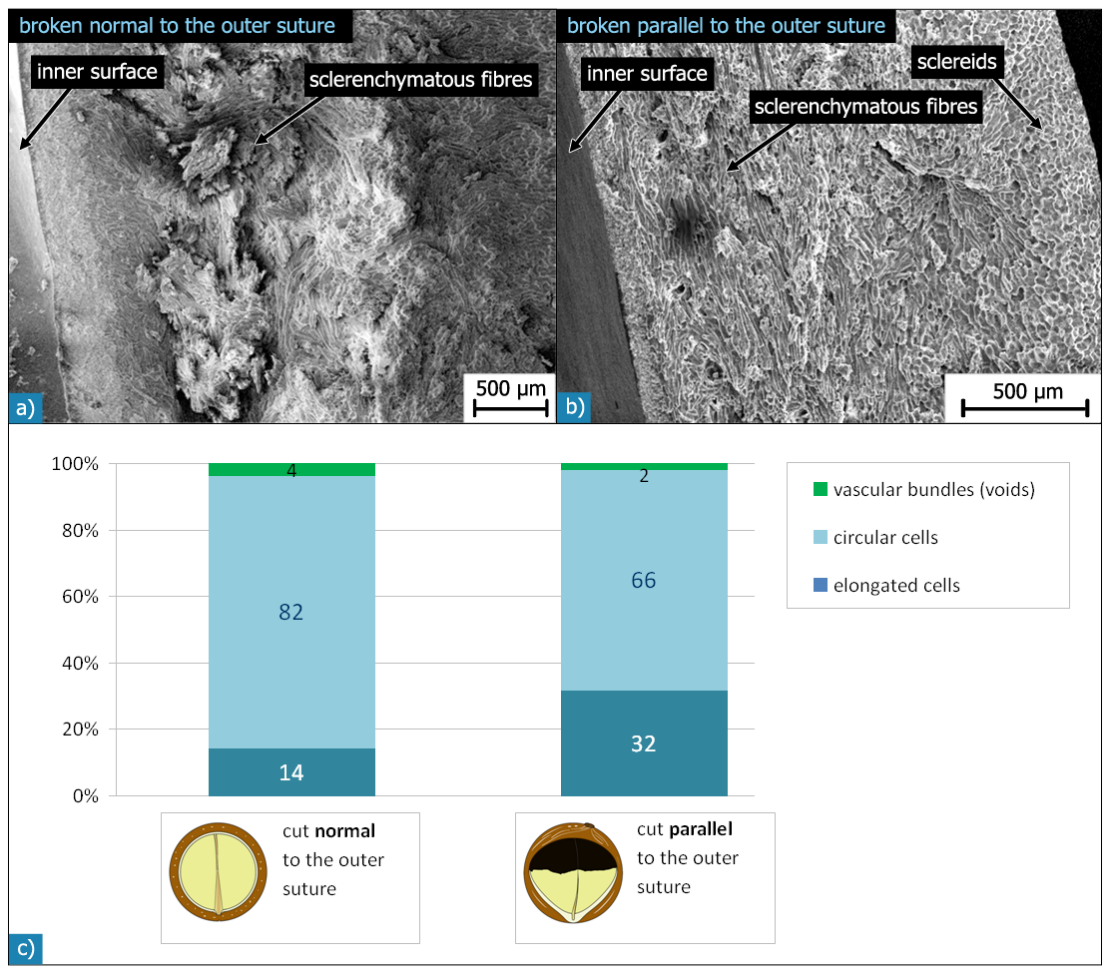
SEM micrographs of *Macadamia* seed coat fracture surfaces broken normal (a) and parallel (b) to the outer suture. The “normal” fracture surface (a) is rougher with many sclerenchymatous fibres protruding at different angles. The “parallel” fracture surface (b) is smoother because sclerenchymatous fibres are mainly orientated parallel to the fracture surface. The diagrams in c) show the area fractions of different cell shapes within the sclerenchymatous tissue for sections cut normal or parallel to the outer suture.

SEM investigations of different fracture surfaces show that shells, fractured parallel to the outer suture, exhibited more fibre bundles running parallel to the suture (hilum-micropyle direction) than shells fractured normal to the outer suture (*fig. 9a & b*). This suggests a preferred orientation of a majority of the sclerenchymatous fibre bundles running curvilinear with the shell’s contour from the hilum to the micropyle. The remaining bundles of sclerenchymatous fibres cross or entwine the orientated fibre bundles so that a compact network with a preferred orientation of fibre bundles is formed. This assumption was validated by a quantitative analysis of light micrographs of several sections, which were cut parallel or normal to the outer suture. The sections cut parallel to the outer suture exhibit more than double the amount of elongated cells (32%) than those found in sections cut normal to the outer suture (14%) (*fig. 9c*). While distinctly elongated cells in the sections are clearly related to sclerenchymatous fibres, the classification of cells with a circular shape is not at all clear: they could be spherical sclereids or sclerenchymatous fibres cut orthogonal to their fibre axis. Thus, the area fractions do not represent the real ratio of fibres and sclereids in the shell. Nevertheless, the difference observed in the relative amount of circular cells for differently oriented sections suggests that there is a preferred orientation of the elongated cells: in one direction, they are cut more or less parallel to their long axis, while in the orthogonal direction, they are cut more or less normal to their long axis and appear as circular features in these sections. Thus the sclerenchymatous fibres have a preferred orientation in the hilum-micropyle direction. In some regions of the shell, the sclerenchymatous fibre layer (L3) is followed by another, relatively thin (35 µm) sclereid layer (L4).

**Figure 10:**
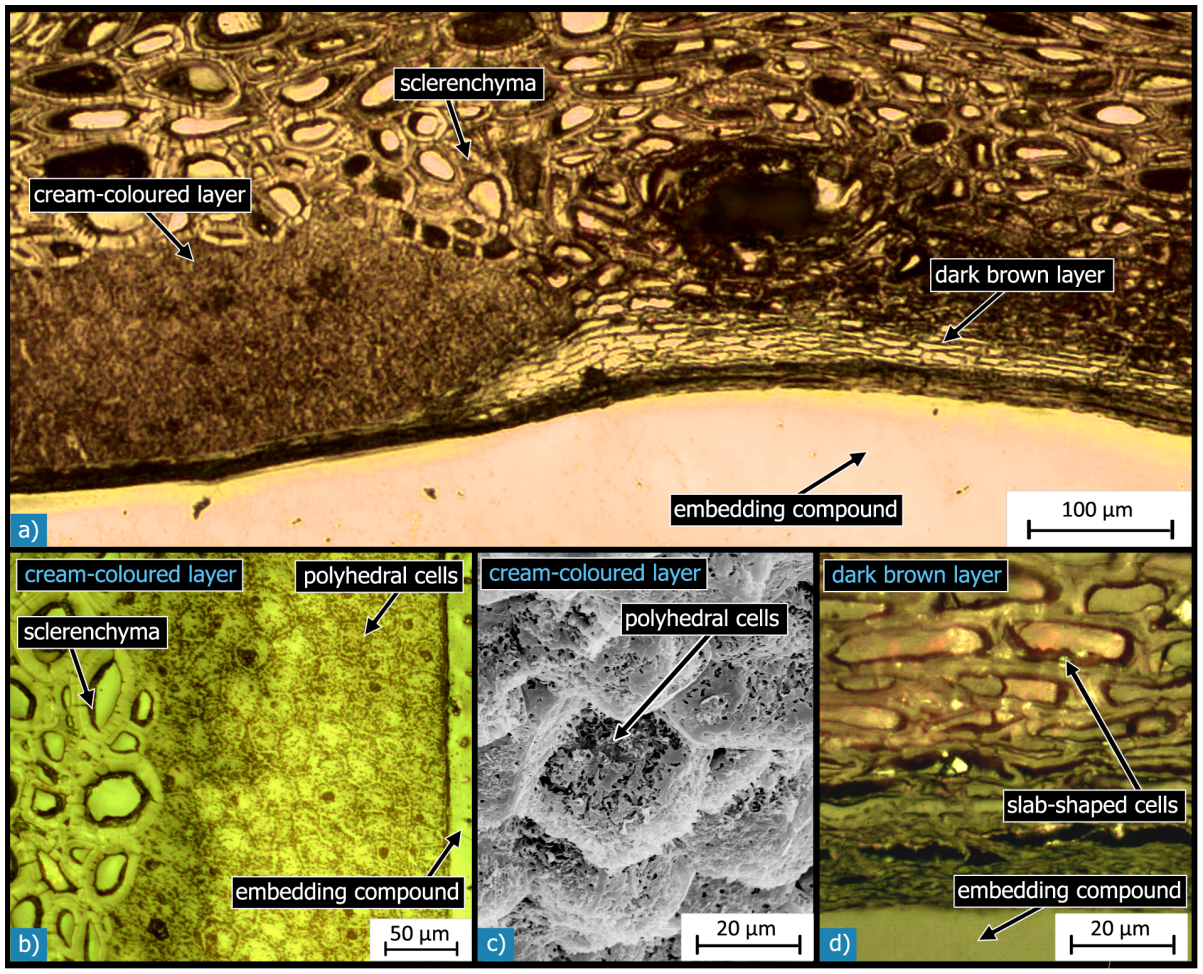
Microstructure of the inner layers and the interfaces between them and to the adjacent layers: Light micrographs of polished sections show the sclerenchymatous and the cream-coloured (L5.1; a, b) and dark brown (L5.2; a, d) inner layers and the interfaces between the different layers. The cream-coloured layer (a, b) is composed of polyhedral cells with thin cell walls. The SEM micrograph in c) of a fracture surface shows the fine and fibrous microstructure of the cells in the cream-coloured layer. The light micrograph in d) shows the dark brown layer, which is composed of slap-shaped cells with thickened cell walls.

The cream-coloured layer on one half of the inside of the shell (L5.1) is composed of non-sclerenchymatous tissue, as can be seen in the micrographs in *fig. 10b & c*. Therefore, it is relatively soft in comparison with the rest of the seed coat material. The other half is covered by a dark brown layer (L5.2), which is composed of slab-shaped cells (*fig. 10a & d*). The cream-coloured layer (L5.1) is on average 170 µm thick which is about three times the thickness of the dark brown layer (L5.2; 55 µm). Both layers (L5.1 and L5.2) are strongly interlocked with the adjoining sclerenchyma (*fig. 10a*). The inner testa layer (L6) is much thinner than the epidermis - its thickness is in the range of 1 to 2 µm – and exhibits no pores (*fig. 6c & d*).

Other features on this hierarchical level are the vascular bundles, visible as white structures that may be observed in photographs of cut seed coats (*fig. 3c & d*). They originate at the hilum and are most abundant in the volume nearby. These bundles have diameters of up to 500 µm and exhibit many branches and interconnections to other bundles. Tomographic investigations revealed that they run through the whole seed as a network with decreasing density from the hilum to the micropyle and split up into smaller bundles (*fig. 3e & f*) [*8*]. Except very close to the hilum, the bundles are oriented with their long axis parallel to the outer and inner sutures, and they are more abundant in the shell volume towards the inside of the seed coat. Thus, a higher number of these structures can be seen in sections cut parallel to the outer suture, while the structures show a more circular shape when the shell is cut normal to the outer suture.

### Hierarchical level H5

**Figure 11:**
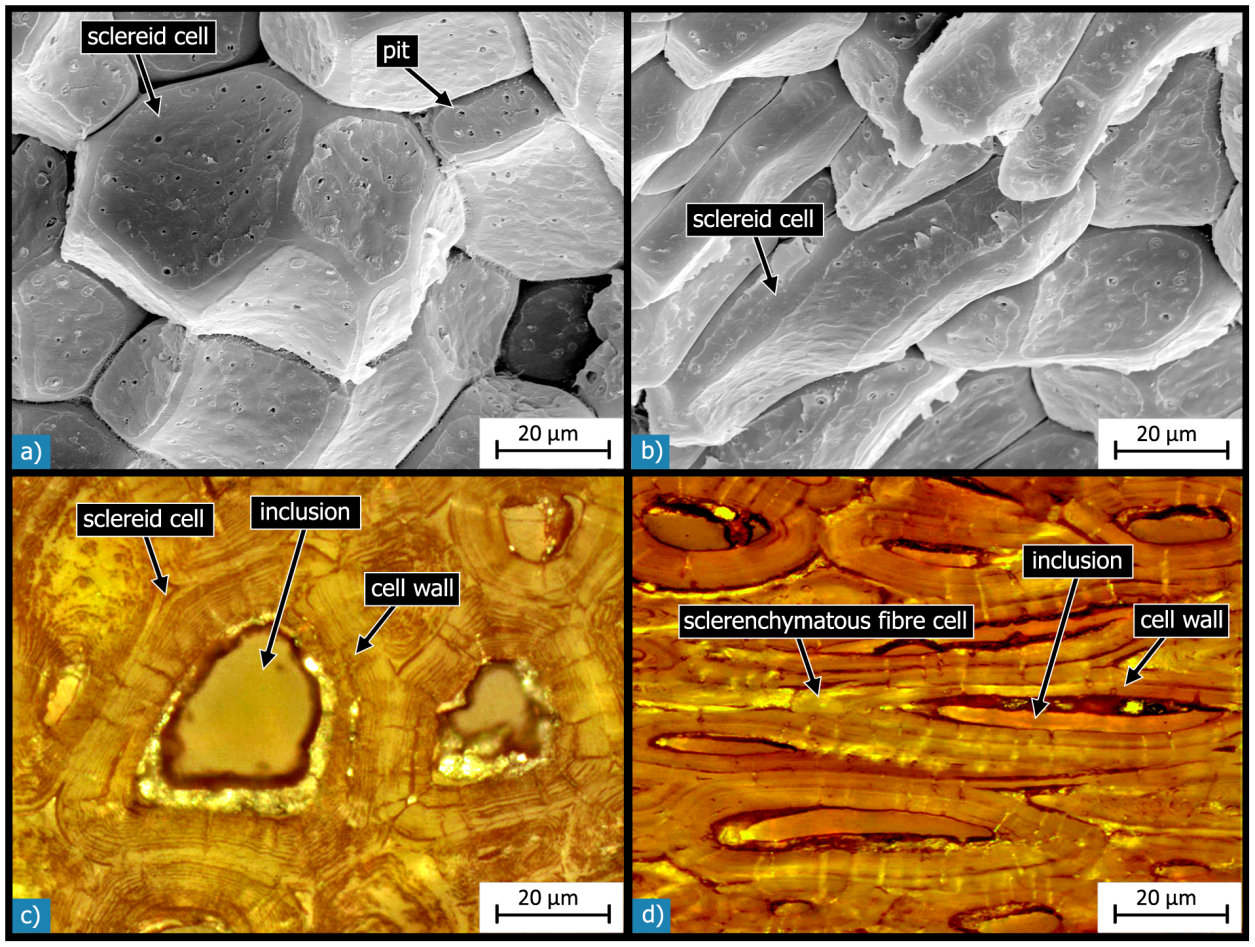
Microstructure of the cells: SEM micrographs of cells in the outer sclereid layer show that they have an isodiametric shape near the outer surface (a) and a more and more ellipsoidal shape with increasing distance from the shell’s outer surface (b). Light micrographs of polished sections show the structural composition of sclereid cells (c) and of sclerenchymatous fibre cells (d), which have a similar microstructure. Both types of cells have thickened and lignified cell walls and a less dense inclusion within their lumen.

There are four different types of cells (*fig. 11* *)*, which vary in shape, dimension and composition. The cell dimensions were estimated by evaluating the light and SEM micrographs (*table 2*). Most of the *Macadamia* seed coat is made up of sclerenchymatous cells, that is sclereid cells and sclerenchymatous fibres (*fig. 5* *&* *8*).

The sclereid cells are almost always isodiametric in the outer shell region, and they become more and more ellipsoidal, dumbbell- or kidney-shaped with increasing distance from the outer testa surface (*fig. 11a & b*). The ratio of the biggest to the smallest cell dimension ranges up to 3∶1. The sclereids have diameters of between 20 to 50 µm. Every sclereid cell is connected to approximately 10 to 12 neighbouring cells via their outer cell wall layers. The locally existing inner sclereid layer (L4) consists of about two to eight cell layers of ellipsoidal, dumbbell- or kidney-shaped sclereids having the same size and structure as the sclereids in the outer sclereid layer (L2) (*fig. 8c*).

The sclerenchymatous fibres are arranged in bundles. Within the bundles every fibre cell is enclosed by approximately four to eight neighbouring cells and is connected to them via the outer cell wall layers. The length of the individual fibrous cells is in the range of several hundreds of microns. However the intertwined arrangement of the individual fibres makes it difficult to analyse the fibre length exactly. Their cross-sectional diameter is a bit smaller than that of the sclereid cells, in the range of 10 to 30 µm.

The flat pancake-like cells of the epidermis (L1) have a thickness of 5 to 10 µm and diameters of approximately 20 to 40 µm. The cells of the cream-coloured layer (L5.1) have a polyhedral shape with approximately 10 to 14 faces. Their diameter spans 20 to 30 µm and their length is about 30 to 40 µm (*fig. 10b & c*). The dark brown layer (L5.2) is composed of slab-shaped cells with diameters between 4 and 5 µm and lengths in the range of 20 to 35 µm (*fig. 10d*).

**Figure 12:**
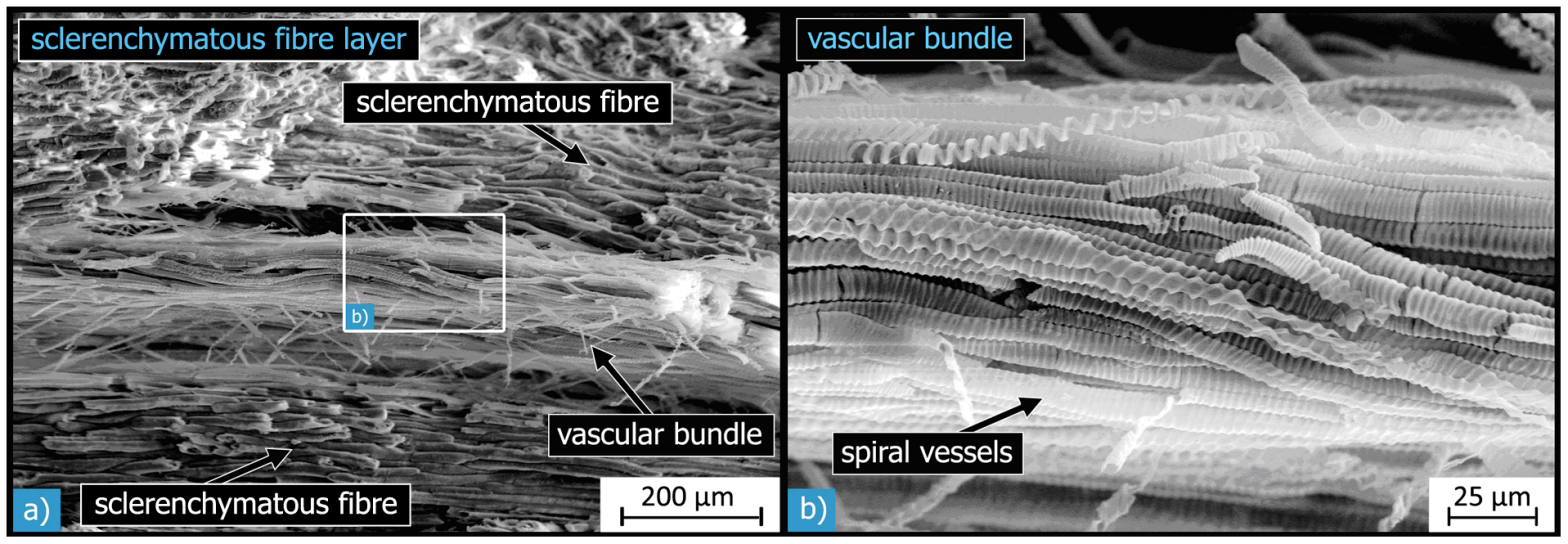
SEM micrographs of vascular bundles within the sclerenchymatous fibre layer: the vascular bundles are surrounded by sclerenchymatous fibres (a). Each bundle consists of many tube-like cells, so-called spiral vessels and tracheids (b).

The vascular bundles are composed of entwined hollow tubes (*fig. 12*). In the vascular bundles, a high number of so-called spiral vessels and tracheids are densely arranged parallel with one another. Their diameter is between 8 to 12 µm, and their walls seem to have circumferential stiffeners in a helical arrangement.

### Hierarchical level H6

This level relates to the structure of the biological cells in the single layers of the sandwich. The sclereids and the sclerenchymatous fibres show a similar composition with strong thickened and lignified cell walls (*fig. 11c & d*). The sclereid cell walls are between 7 and 15 µm thick, while the cell walls of the sclerenchymatous fibres are a bit thinner (7 to 10 µm). In the lumen of both types of cells there is a less dense inclusion, presumably calcium oxalate [*20*] or lignin [11], [12].

The cell walls of the sclereids and the sclerenchymatous fibres contain many pits (*fig. 11a*), which act as connections to neighbouring cells for fluid, nutrient and signal exchange during cell development when the cells are still alive [26]. The diameter of the pits is in the range of 0.1 to 0.2 µm. The pits can be seen as fine shiny lines under the light microscope.

The slab-shaped cells of the dark brown layer are about 1 to 2 µm thick. The polyhedral cells of the cream-coloured inner layer show a fine and fibrous structure (*fig. 10b & c*). Light micrographs of polished sections further revealed some spherical components inside these cells. The cell walls are about 1 µm thick.

### Hierarchical level H7

This is defined as the structure of the individual cell components such as the cell walls. The walls of sclerenchymatous cells often show thickened and lignified secondary cell walls, which develop after the primary cell wall is complete and the cell growth has stopped. The secondary cell walls of the sclereids and the sclerenchymatous fibres have typical well marked stratifications [25], [26], which have a concentric arrangement around the central lumen and which are transversed by the pit channels (*fig. 11c & d*).

### Hierarchical level H8

In light micrographs, each secondary cell wall stratification has a specific colour. This is assumed to be influenced by different chemical compositions of the individual cell wall layers or by different orientations of the molecular components [26]. Besides lignin, which amounts to about 20 to 35%, the walls of lignified cells typically contain cellulose as main component contributing typically 60 to 70%. The cellulose microfibrils have varying orientations in the various layers of the secondary cell walls. Further constituents are pectin and hemicellulose [26], [27].

## Discussion

Our investigations of *Macadamia integrifolia* seed coats reveal a hierarchical and highly anisotropic structure of this very strong and tough material. In addition to quantitative information (*table 2*) about the coat’s composition and its structural elements, our investigations have shown a more complex structural makeup of the *Macadamia* seed coat (*fig. 2*) than has previously been described. We identified nine hierarchical levels (including the fruit), whereby our classification is different to the one usually applied in biology. This is justified by the mechanical relevance and properties of the entities we defined. For instance, testing a small piece of the testa that corresponds to the hierarchical level H3 is assumed to have another mechanical response than the whole seed coat (H2), which contains the notch-like sutures as well as the hilum and the micropyle. Another example is the differentiation of up to three different layers in the sclerenchymatous tissue (L2–L4) where the cell shape was used as the distinguishing criterion similar to the work of others (e.g. [11]). Even though the shell as a whole is composed of sclerenchymatous cells, the three layers of the sandwich behave very differently regarding crack growth, and we assume that the sandwich arrangement itself is an important factor influencing the toughness and strength of the shell. The hierarchical classification is slightly arbitrary in places, for instance sutures appear on H1 and H2 because of the visibility of the inner sutures. Regarding their size, the inner sutures could also be assigned H1 like the outer suture.

On the level of the whole seed coat (H3), we found a sandwich arrangement of five, or locally six concentric layers. Three of these layers (L2 to L4) belong to the so-called sclerenchymatous tissue and consist of different amounts of two cell-types: isodiametric sclereid cells, and elongated fibre cells. The sclereid layers (L2, L4) and the intermediate sclerenchymatous fibre layer (L3) make up more than 95% of the shell. The results correspond well with previous investigations [11], [19], [21]. However, we did not find any structures in the outer shell region for both the natural and dewaxed states (*fig. 5b*, *6a & b*, *7* *)* that could be identified as “elastic stiffeners”. Kaupp & Naimi-Jamal [19] further describe an “outer hardshell layer which might correspond to what we refer to as the sclerenchyma volume (layers L2 to L4, *fig. 5*) or to the outer sclereid volume (only layer L2) of the shell, and an “inner chewable” layer that might correspond to the sclerenchymatous fibre layer (1270 µm thick) that we saw. However, the sclerenchymatous layers of the shells we investigated proved to be rather homogeneous regarding their Vickers hardness (about 35 HV 0.1 [*7*]).

The observed morphology and dimensions of the sclerenchymatous cells (sclereids and sclerenchymatous fibres) and their arrangement correspond well with the features described in former work [10]–[12], [19], [21], [24]. We distinguished various fibrous structures within the testa of *Macadamia* seeds. These were the sclerenchymatous fibres, their arrangements in bundles, vascular bundles and the single spiral vessels and tracheids. We showed a highly anisotropic arrangement of these elongated structures on different hierarchical levels. This is in agreement with the anisotropic arrangement of fibrous structures described by Kaupp & Naimi-Jamal [19]. However, they saw a different preferred orientation, normal to the outer surface, while the structures we saw are oriented mostly parallel to the outer surface of the shell. Others described the microstructure of *Macadamia* seed coats as an isotropic arrangement of elongated fibres [10], [12]. Our quantitative image analysis revealed a preferred orientation of the majority of the fibre bundles in the direction from the hilum to the micropyle. The rest of the bundles are orientated randomly as previously described. Nevertheless, we are uncertain on how accurate the measurements of the sclerenchymatous fibres are: because the fibres are not fully parallel to each other over their entire length they might not be fully visible and their real length is probably greater than estimated. Furthermore, the evaluation of polished sections may lead to erroneous interpretations because the cell-shape cannot be determined and the 3D-arrangement of fibrous structures cannot be seen properly. In the lab µCT investigations performed so far the resolution is not sufficient to distinguish single cells.

It may be that some of the differences that we see between the results of others and our description may come from “inter-species” differences. While most groups [10], [19], [22], [23] investigated *Macadamia ternifolia*, only two groups [20], [21] examined the microstructure of *Macadamia integrifolia* as we did. However, confusion exists in the literature as to what extent these species are different: several authors point out that *Macadamia integrifolia* is a variety of *Macadamia ternifolia* [20], [28].

**Figure 13:**
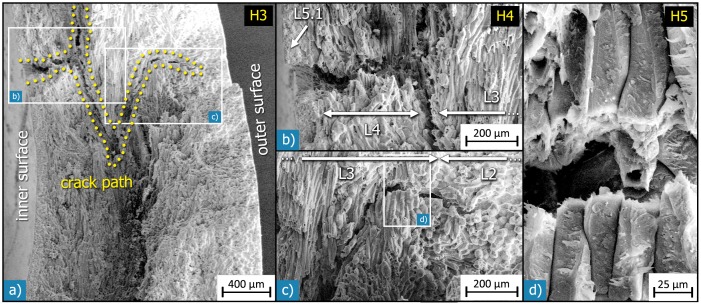
SEM micrographs showing a deflected and branched crack path on different length-scales: a) fracture surface of an entire *Macadamia* seed coat after loading in compression; crack deflection took place in three dimensions, as the topography of the fracture surface and the secondary cracks visible on it show. A magnified view of the crack part near the inner surface shows that the crack was deflected at the interface between the inner sclereid layer (L4) and the sclerenchymatous fibre layer (L3). c) The secondary crack stops at the interface between the sclerenchymatous fibre layer (L3) and the outer sclereid layer (L2). d) In the main fracture surface, the crack mostly follows the interfaces between the cells; in this area, however, the secondary crack fractured some sclerenchymatous fibres vertical to their long axis.

The *Macadamia integrifolia* hierarchical levels and their main building blocks interact and result in astounding mechanical properties of the seed coat. The strength of these seed shells is much higher than the strength of comparable shells (see *fig. 1* & *table 1*, [7]–[18]). Further, toughness is also high, as seen under uniaxial compression, where the whole seeds usually show remarkable behaviour: even after formation of a clearly visible macroscopic crack, the load does not instantaneously decrease to zero and surprisingly the seed is still able to carry load, as seen from the jagged force-displacement curve [7], [13]. Similar observations were made in tension and compression tests on mm-sized specimens machined from the sclerenchyma layer [8]. These findings hint to an intermittent crack growth behaviour [8], [13]. Different microstructural mechanisms increase the fracture energy. These include crack deflection at the interfaces of the sandwich layers (H3, e.g. *fig. 13c*), crack deflection and branching by the vascular bundles (H4) [8] and intercellular crack growth within the sclerenchymatous layers (H4, H5). These mechanisms lead to extended crack paths and thus result in an increase in toughness (*fig. 13*). Further toughening might be due to the almost spherical shape of the seed (H1/H2) [19] or be influenced by the mechanical behaviour of the secondary cell walls (H7) and their interfaces. The hierarchical structure and the interaction of the different hierarchical levels thus greatly affect the macroscopic mechanical behaviour of the seed. The knowledge of the complex microstructure thus provides the basis to understand and quantify these structure-function relationships.

Finally, we discuss a question relevant from a biological viewpoint: why do the seeds need to be so strong? Detailed knowledge of the microstructure may help to identify the types of loading and or abrasion that the seed coat faces when protecting the seed, although the intricate structure may have formed due to other reasons. In order to elucidate the ecological significance of the extraordinarily hard and tough seed coats of *Macadamia* and to understand the selective pressures causing such a structure, seed dispersal strategies and predators native to the original habitat of the plant have to be taken into consideration. Unfortunately, only little is known about the dispersal of *Macadamia* seeds in natural habitats. Different vectors such as water, gravity and animals have been proposed as being important for the dispersal of *Macadamia* seeds [29], [30]. Recent studies support the idea that the seeds are mainly dispersed by gravity and flooding events. However, the potential importance of the latter mechanism is biased by the fact that mature *Macadamia* seeds do not float [30], [31]. Nowadays, rodents *(Rattus rattus, Uromys caudimaculatu)* seem to be the most important animal vectors in orchards in Australia [30], [32], [33]. However, as most *Macadamia* seeds found in rat burrows are damaged [34] and - most importantly - as rats are not native to Australia, the dispersal by rodents cannot have been selectively decisive for the evolution of the hard and tough seed shell of *Macadamia*. As to the importance of other animals (birds, other mammals or marsupials) as vectors in the natural habitat, only little information exists [29], [35]. The best conceivable selective pressures acting on the evolution of such a mechanically resistant seed coat may be mechanical impact due to rolling over ground during heavy flooding events, and feeding or egg laying insects (beetles) which are able to penetrate nearly any type of known fruit or seed coat by their mouthparts or ovipositors [36]. We hypothesize that the strength of the *Macadamia* seed coat is primarily necessary as protection against abrasive transport over ground, and that the structure of *Macadamia integrifolia* is well suited to fulfil this function.
